# Comparison between OK-432 and Talc for pleurodesis in patients with persistent pulmonary air leak: a Japanese nationwide retrospective database study

**DOI:** 10.1007/s11748-024-02088-w

**Published:** 2024-09-26

**Authors:** Jumpei Taniguchi, Shotaro Aso, Taisuke Jo, Hiroki Matsui, Kiyohide Fushimi, Hideo Yasunaga

**Affiliations:** 1 Department of Clinical Epidemiology and Health Economics School of Public Health, The University of Tokyo, 7-3-1, Hongo, Bunkyo-ku, Tokyo 113-8655 Japan; 2 Department of Real World Evidence, Graduate School of Medicine, The University of Tokyo, 7-3-1, Hongo, Bunkyo-ku, Tokyo 113-8655 Japan; 3 Department of Health Policy and Informatics, Graduate School of Medical and Dental Sciences, Tokyo Medical and Dental University, 1-5-45 Yushima, Bunkyo-ku, Tokyo 113-8510 Japan

**Keywords:** OK-432 (Picibanil^®^), Pleurodesis, Pneumothorax, Talc, Treatment outcome

## Abstract

**Objectives:**

OK-432 (Picibanil^®^) and talc are used in patients with persistent pulmonary air leaks. However, it is unclear which of these two agents is more effective.

**Methods:**

This retrospective study used data from the Japanese Diagnosis Procedure Combination inpatient database. Patients with pneumothorax who underwent chemical pleurodesis between July 2010 and March 2022 were included in this study. The patients were categorized into two groups: the OK-432 and talc groups. The primary outcome measure was treatment failure, defined as a composite of requirement for additional surgical procedures, bronchoscopic interventions, or chemical pleurodesis. The secondary outcome measures were in-hospital mortality, length of hospital stay, 30-day readmission, and incidence of interstitial lung diseases after hospitalization. To compare the outcomes between the groups, 1:4 propensity score matching was conducted.

**Results:**

Among the 4179 eligible patients, 3551 and 628 patients underwent chemical pleurodesis using OK-432 and talc, respectively. Propensity score matching yielded 2508 and 627 patients who underwent chemical pleurodesis using OK-432 and talc within seven days of admission, respectively. The frequency of treatment failure in the talc group (37.5% vs. 31.4%; *P* = 0.006) was lower than that in the OK-432 group with no significant differences in other outcomes.

**Conclusions:**

Medical professionals can consider talc as the initial pleurodesis agent for patients with persistent air leaks.

**Supplementary Information:**

The online version contains supplementary material available at 10.1007/s11748-024-02088-w.

## Introduction

Persistent air leakage is frequently observed in patients with pneumothorax caused by an underlying lung disease (secondary spontaneous pneumothorax), pulmonary infections, complications of mechanical ventilation, chest trauma, or pulmonary surgery [1]. Persistent air leaks can result in complications, such as pneumonia, empyema, and venous thromboembolism, readmission to the intensive care unit ICU, and prolonged hospital stays [2]. Surgical repair remains the gold standard of treatment [3]. However, it may not be an appropriate choice for elderly patients who are unfit to undergo surgery or those who cannot undergo surgical interventions.

Chemical pleurodesis is a less-invasive treatment option compared with surgical intervention for persistent air leaks [4] that induces an inflammatory response, leading to the formation of scar tissue that obliterates the pleural space and seals the pleural defect [2]. Agents such as talc, tetracycline derivatives, OK-432 (Picibanil^®^), autologous blood patch, and 50% glucose solution have been used in chemical pleurodesis [4]. OK-432 was approved as a pleurodesis agent for malignant pleural effusion in Japan before talc, leading to its more frequent use [5]. OK-432 and talc have sometimes been used off-label for pneumothorax treatment, due to their suggested effectiveness [1, 4]. The success rates of chemical pleurodesis in patients with persistent air leaks range from 60–90% [2, 6]. However, it is unclear which of these agents is more effective in patients with persistent air leaks. Therefore, this study retrospectively evaluated the effectiveness of talc and OK-432 in patients with persistent air leaks using a Japanese national inpatient database.

## Material and methods

### Data Source

This study was approved by the Institutional Review Board of the University of Tokyo (3501-(5), May 19th, 2021). The requirement for obtaining informed consent was waived owing to the retrospective nature of the study and the anonymized nature of the data.

We used data from the Japanese Diagnosis Procedure Combination inpatient database. The database included patient demographic data such as age, sex, body height, and body weight; primary diagnosis; comorbidities at the time of admission; post-admission complications during hospitalization (based on the International Classification of Diseases, 10th revision [ICD-10] codes); hospital identification number; dates of initiating therapy, procedures, and surgery; activities of daily living score; the Japan Coma Scale score at the time of admission; discharge status (deceased or alive); and dates of hospital admission and discharge [7]. The database also contains the discharge abstracts and administrative claims data from over 1200 hospitals across Japan [8]. The validity of the database has been demonstrated in a previous study, which revealed a diagnostic specificity and sensitivity of > 96% and 50–80%, respectively. The specificity and sensitivity of the procedures were > 90% [9].

### Patient data

Patients hospitalized for pneumothorax (ICD-10 codes: J93, N948, and T812) between July 2010 and March 2022 were identified from the database. Only the data from the first hospitalization were included in the case of patients who were hospitalized more than once during the study period. Pregnant women, patients who were discharged or died within seven days after admission, those aged < 15 years, those who had undergone a surgical procedure before or on the same day as pleurodesis, those with traumatic pneumothorax, and those who had undergone pleurodesis using both OK-432 and talc on the same day were excluded. Those who did not undergo pleurodesis within seven days after admission were excluded because only an air leak lasting for more than 5–7 days was considered a persistent pulmonary air leak [1].

The patients were divided into two groups: those who had undergone pleurodesis using OK-432 within seven days after admission (OK-432 group) and those who had undergone pleurodesis using talc within seven days after admission (talc group).

### Study variables

Patient characteristics included age; sex; body mass index on admission; smoking history (nonsmoker, current/past smoker); Japan Coma Scale score at the time of admission; Charlson comorbidity index [10]; Barthel index [11]; comorbidities (chronic obstructive pulmonary disease, asthma, interstitial pneumonia, fungal lung disease, bronchiectasis and nontuberculous mycobacteria, empyema, lung cancer, chronic respiratory failure, liver failure, diabetes mellites, autoimmune disease, kidney failure, cardiovascular disease, and dementia); admission to teaching hospital; admission to the intensive or high care unit; and treatments (oxygenation, mechanical ventilation, renal replacement therapy, use of antibiotics, antifungal drugs, and steroid therapy) within two days after admission. Supplementary Table 1 presents the comorbidities at the time of admission identified using the ICD-10 codes.

The patients were categorized into four groups based on the body mass index: < 18.5 kg/m^2^, 18.5–24.9 kg/m^2^, 25.0–29.9 kg/m^2^, and ≥ 30.0 kg/m^2^. The Japan Coma Scale score was converted to the Glasgow Coma Scale score based on a previous report [12]. A substantial correlation has been observed between the Japan Coma Scale and the Glasgow Coma Scale [13]. The patients were categorized into four groups based on the Barthel Index: 0, 5–50, 55–95, and 100. The patients were categorized into five groups based on the Charlson comorbidity index scored according to the diagnosis of each patient: 0, 1, 2, 3, or ≥ 4 [10].

### Outcome measurement

The primary outcome measure was treatment failure. Treatment failure was defined as the composite outcome of the requirement for additional surgical procedures, chemical pleurodesis, and endobronchial occlusion using silicone spigots.

The secondary outcome measures were in-hospital mortality, length of hospital stay, readmission within 30 days after discharge, and incidence of interstitial lung diseases after hospitalization (ICD-10 codes: J702, J703, J704, J80, and J84).

### Statistical analysis

Propensity score matching was performed to compare the outcomes between the OK-432 and Talc groups [14]. A multivariate logistic regression model was used to predict the propensity scores for undergoing chemical pleurodesis using talc. The predictor variables are shown in the baseline characteristic table. Subsequently, 1:4 nearest neighbor matching was performed after replacing the estimated propensity scores using a caliper width set at 20% of the standard deviation for the propensity scores [14, 15]. The baseline characteristics before and after propensity score matching were compared using absolute standardized differences. Absolute standardized differences of ≤ 10% between the OK-432 and talc groups were considered negligible imbalances [16]. Propensity score matching was performed using the STATA module of PSMATCH2 provided by Leuven and Sianesi [17].

Crude outcomes in the unmatched cohort were compared using t-test for continuous variables and the chi-square test for categorical variables. A generalized estimating equation approach was used in the score-matched cohort to compare the primary and secondary outcomes accompanied by cluster-robust standard errors, with individual hospitals as clusters [18]. Risk differences and 95% confidence intervals (CIs) for the primary and secondary outcomes were calculated subsequently.

Continuous variables are presented as means with standard deviations, whereas categorical variables are presented as numbers (%). All *P* values are two-sided, and *P* values < 0.05 were considered statistically significant. All analyses were performed using STATA/MP 17.0 software (StataCorp, College Station, TX, USA).

### Sensitivity analyses

We performed a sensitivity analysis including patients hospitalized for pneumothorax between January 2014 and March 2022, considering that talc became usable for pleurodesis in Japan in December 2013. We performed another sensitivity analysis excluding patients with lung tumors because, in them, OK-432 or talc might have been used for malignant pleural effusion rather than pneumothorax. We also assessed the E value based on the results of the main primary outcome, which is an effect size used to assess the potential impact of unknown confounders on our observational study results [19].

## Results

**Fig. 1:**
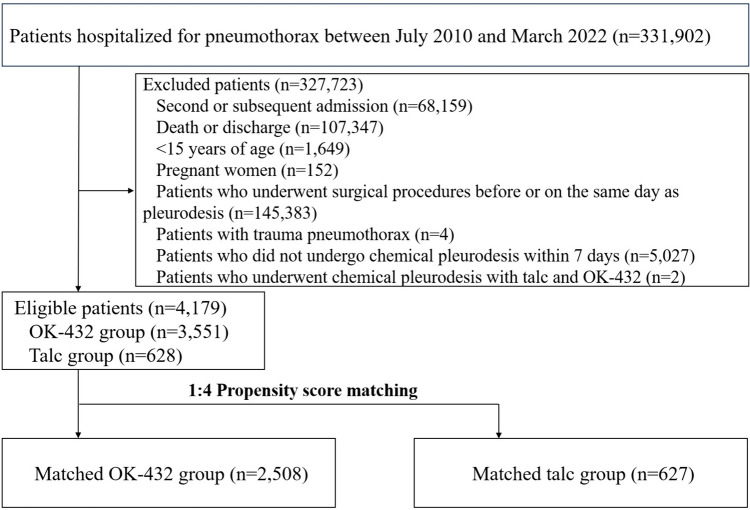
Flowchart of patient selection

In total, 331,902 patients were hospitalized with pneumothorax between July 2010 and March 2022 (Fig. 1). Among them, 4179 patients, comprising 3551 and 628 patients who underwent chemical pleurodesis using OK-432 and talc within seven days of admission, respectively, met the inclusion criteria.

Propensity score matching yielded 2508 and 627 score-matched patients. Table 1 presents the baseline characteristics of the unmatched and propensity-score-matched groups. The patients who underwent chemical pleurodesis using talc were more likely to be female and older than those who underwent chemical pleurodesis using OK-432. The Charlson Comorbidity Index of the talc group was higher than that of the OK-432 group. The presence of comorbidities, such as interstitial pneumonia, lung cancer, and diabetes mellitus, and steroid use was higher in the talc group than that in the OK-432 group.

**Table 1:** Patient characteristics at the time of admission

Variables	Unmatched			Matched		
	OK-432 (n = 3,551)	Talc (n = 628)	ASD	OK-432 (n = 2,508)	Talc (n = 627)	ASD
Age, years, mean (SD)	71.6 (12.9)	74.2 (11.1)	20.9	74.1 (11.5)	74.2 (11.2)	0.6
Male, n (%)	3126 (88.0%)	521 (83.0%)	14.4	2,080 (82.9%)	520 (82.9%)	0.0
BMI, kg/m^2^, n (%)						
< 18.50	1312 (36.9%)	198 (31.5%)	11.4	787 (31.4%)	198 (31.6%)	0.4
18.50–24.99	1721 (48.5%)	301 (47.9%)	1.1	1220 (48.6%)	301 (48.0%)	1.3
25.00–29.99	212 (6.0%)	49 (7.8%)	7.2	192 (7.7%)	49 (7.8%)	0.6
≥ 30.00	17 (0.5%)	2 (0.3%)	2.5	5 (0.2%)	2 (0.3%)	2.4
Missing data	289 (8.1%)	78 (12.4%)	14.1	304 (12.1%)	77 (12.3%)	0.5
Smoking history, n (%)						
Nonsmoker	980 (27.6%)	182 (29.0%)	3.1	763 (30.4%)	182 (29.0%)	3.1
Current/past smoker	2164 (60.9%)	374 (59.6%)	2.8	1,466 (58.5%)	373 (59.5%)	2.1
Missing data	407 (11.5%)	72 (11.5%)	0.0	279 (11.1%)	72 (11.5%)	1.1
GCS score on admission, mean (SD)	14.9 (1.0)	14.8 (1.0)	2.8	14.9 (0.9)	14.8 (1.0)	4.9
Barthel index on admission, n (%)						
0	291 (8.2%)	55 (8.8%)	2.0	228 (9.1%)	55 (8.8%)	1.1
5–50	502 (14.1%)	80 (12.7%)	4.1	325 (13.0%)	80 (12.8%)	0.6
55–95	772 (21.7%)	119 (18.9%)	6.9	474 (18.9%)	119 (19.0%)	0.2
100	1507 (42.4%)	257 (40.9%)	3.1	1030 (41.1%)	257 (41.0%)	0.2
Missing	479 (13.5%)	117 (18.6%)	14.0	451 (18.0%)	116 (18.5%)	1.3
Charlson comorbidity index, n (%)						
0	948 (26.7%)	123 (19.6%)	16.9	563 (22.4%)	123 (19.6%)	7.0
1	1153 (32.5%)	134 (21.3%)	25.3	551 (22.0%)	134 (21.4%)	1.5
2	563 (15.9%)	140 (22.3%)	16.4	454 (18.1%)	140 (22.3%)	10.5
3	432 (12.2%)	64 (10.2%)	6.3	270 (10.8%)	64 (10.2%)	1.8
≥ 4	455 (12.8%)	167 (26.6%)	35.2	670 (26.7%)	166 (26.5%)	0.5
Lung disease, n (%)						
Chronic obstructive pulmonary disease	1450 (40.8%)	197 (31.4%)	19.8	772 (30.8%)	197 (31.4%)	1.4
Asthma	192 (5.4%)	26 (4.1%)	5.9	96 (3.8%)	26 (4.1%)	1.6
Interstitial pneumonia	273 (7.7%)	77 (12.3%)	15.3	325 (13.0%)	76 (12.1%)	2.5
Fungal lung disease	32 (0.9%)	6 (1.0%)	0.6	18 (0.7%)	6 (1.0%)	2.6
Bronchiectasis & NTM of the lungs	32 (0.9%)	6 (1.0%)	0.6	19 (0.8%)	6 (1.0%)	2.2
Empyema	11 (0.3%)	5 (0.8%)	6.6	24 (1.0%)	5 (0.8%)	1.7
Lung cancer	591 (16.6%)	138 (22.0%)	13.5	553 (22.0%)	138 (22.0%)	0.1
Chronic respiratory failure	233 (6.6%)	43 (6.8%)	1.1	191 (7.6%)	42 (6.7%)	3.6
Cardiovascular disease, n (%)	209 (5.9%)	40 (6.4%)	2.0	162 (6.5%)	40 (6.4%)	0.3
Kidney failure, n (%)	61 (1.7%)	11 (1.8%)	0.3	62 (2.5%)	11 (1.8%)	5.0
Liver failure, n (%)	47 (1.3%)	12 (1.9%)	4.7	42 (1.7%)	12 (1.9%)	1.8
Diabetes mellitus, n (%)	439 (12.4%)	108 (17.2%)	13.7	433 (17.3%)	107 (17.1%)	0.5
Autoimmune disease, n (%)	63 (1.8%)	10 (1.6%)	1.4	41 (1.6%)	10 (1.6%)	0.3
Dementia, n (%)	104 (2.9%)	27 (4.3%)	7.3	106 (4.2%)	27 (4.3%)	0.4
ICU or HCU admission, n (%)	93 (2.6%)	17 (2.7%)	0.5	91 (3.6%)	17 (2.7%)	5.2
Teaching hospital admission, n (%)	3030 (85.3%)	544 (86.6%)	3.7	2,158 (86.0%)	543 (86.6%)	1.6
Treatment within two days of admission day, n (%)						
Oxygenation	1,989 (56.0%)	347 (55.3%)	1.5	1,337 (53.3%)	346 (55.2%)	3.8
Mechanical ventilation	90 (2.5%)	17 (2.7%)	1.1	72 (2.9%)	17 (2.7%)	1.0
Renal replacement therapy	13 (0.4%)	0 (0.0%)	8.6	0 (0.0%)	0 (0.0%)	0.0
Antibiotics	926 (26.1%)	157 (25.0%)	2.5	667 (26.6%)	156 (24.9%)	3.9
Antifungal drugs	28 (0.8%)	9 (1.4%)	6.2	23 (0.9%)	9 (1.4%)	4.8
Steroids	397 (11.2%)	95 (15.1%)	11.7	385 (15.4%)	94 (15.0%)	1.0

*ASD* absolute standardized difference, *BMI* body mass index, *GCS* Glasgow Coma scale, *HCU* high care unit, *ICU* intensive care unit, *NTM* nontuberculous mycobacteria, *SD* standard deviation

The total number of etiologies does not add up to 100% as more than one cause was assigned to a single patient

Table 2 presents the crude outcomes of the unmatched cohort. Significant differences were observed between the two groups in terms of treatment failure (35.6% vs. 31.5%, *P* = 0.047), in-hospital mortality (6.0% vs. 8.8%, *P* = 0.010), and readmission within 30 days of discharge (5.6% vs. 7.8%, *P* = 0.032). However, the length of hospital stay and incidence of interstitial lung disease after hospitalization did not differ between the groups.

**Table 2:** Comparison of crude outcomes between the two groups

	OK-432 (n = 3,551)	Talc (n = 628)	*P*
Primary outcome			
Treatment failure, n (%)	1,265 (35.6%)	198 (31.5%)	0.047
Surgical procedure after chemical pleurodesis	250 (7.0%)	32 (5.1%)	0.073
Bronchial intervention after chemical pleurodesis	22 (0.6%)	9 (1.4%)	0.029
Additional chemical pleurodesis	1,113 (31.3%)	183 (29.1%)	0.271
Secondary outcome			
In-hospital mortality, n (%)	214 (6.0%)	55 (8.8%)	0.010
Length of hospital stay, days, mean (SD)	18.9 (18.5)	19.3 (17.0)	0.600
Readmission within 30 days, n (%)	199 (5.6%)	49 (7.8%)	0.032
Incidence of interstitial lung diseases, n (%)	23 (0.6%)	4 (0.6%)	0.975

*SD* standard deviation

Almost all baseline characteristics were well-balanced after propensity score matching (Table 1). Table 3 presents the outcomes of the propensity score-matched cohort. The frequency of treatment failure (37.5% vs. 31.4%; risk difference, -6.8%; 95% CI − 11.7 to − 1.9%; *P* = 0.006) and additional pleurodesis use (33.7% vs. 29.0%; risk difference, − 5.4%; 95% CI − 10.2 to − 0.6%; *P* = 0.027) in the talc group was lower than that in the OK-432 group. No significant differences were observed between the two groups in terms of in-hospital mortality, length of hospital stay, readmission within 30 days, or incidence of interstitial lung disease after hospitalization.

**Table 3:** Comparison of outcomes between the groups in the propensity score matched cohort

	OK-432 (n = 2,508)	Talc (n = 627)	Risk difference	*P*
Primary outcome				
Treatment failure, n (%)	940 (37.5%)	197 (31.4%)	− 6.8 (− 11.7 to − 1.9)	0.006
Surgical procedure after pleurodesis use	173 (6.9%)	32 (5.1%)	− 2.1 (− 4.3 to 0.2)	0.074
Bronchial intervention after pleurodesis use	14 (0.6%)	9 (1.4%)	1.0 (− 0.1 to 2.2)	0.082
Additional pleurodesis use	845 (33.7%)	182 (29.0%)	− 5.4 (− 10.2 to − 0.6)	0.027
Secondary outcome				
In-hospital mortality, n (%)	227 (9.1%)	55 (8.8%)	0.2 (− 3.1 to 3.5)	0.902
Length of hospital stay, days, mean (SD)	19.7 (18.7)	19.3 (17.1)	0.0 (− 1.7 to 1.6)	0.968
Readmission within 30 days, n (%)	133 (5.3%)	48 (7.7%)	1.7 (− 1.0 to 4.4)	0.207
Incidence of interstitial lung diseases, n (%)	13 (0.5%)	3 (0.5%)	0.0 (− 0.7 to 0.8)	0.903

*SD* standard deviation

The results of the sensitivity analyses are shown in Supplementary Tables 2 to 5 and are consistent with those in the main analysis. The E value (lower limit of the 95% CI) for the primary outcome was 1.67 (1.30).

## Discussion

This study retrospectively evaluated the effectiveness of talc compared with that of OK-432 in patients with persistent air leaks undergoing chemical pleurodesis for the first time. The frequency of treatment failure and additional pleurodesis was lower in the talc group than that in the OK-432 group. No significant differences were observed between the groups in terms of in-hospital mortality, length of hospital stay, readmission within 30 days, or incidence of interstitial lung disease after hospitalization, indicating that talc may be a more suitable agent than OK-432 for chemical pleurodesis in patients with persistent pulmonary air leaks. Considering the physical burden associated with additional treatments and procedures, physicians can consider performing chemical pleurodesis using talc initially in patients with persistent air leaks.

A major strength of the present study is the use of a nationwide inpatient database. A previous study that compared the outcomes of chemical pleurodesis using OK-432 with those of chemical pleurodesis using talc in patients with persistent air leaks had a small sample size and only included postoperative patients with persistent air leaks from a single center without adjusting for confounding factors [20]. In contrast, the present study included data representing real-world practices for persistent air leaks throughout Japan and used propensity score analyses to reduce bias and strengthen the comparability between the groups.

In Japan, OK-432 has been frequently used off-label in patients undergoing chemical pleurodesis for persistent air leaks. This is because, in Japan, talc was approved after OK-432 for the indication of chemical pleurodesis [5]. A previous single-center retrospective study revealed no significant differences between the outcomes of postoperative patients with persistent air leaks who underwent chemical pleurodesis using OK-432 and talc [20]. An optimal approach or agent has not been established for chemical pleurodesis; however, talc is selected more frequently in Europe and the United States based on existing evidence [4, 21–23]. The present study demonstrated that the frequency of treatment failure in the talc group was lower than that in the OK-432 group. The differences between our findings and the previous study’s may be attributed to the larger sample size of our study and the difference in study populations. Our study mainly comprised patients with secondary spontaneous pneumothorax with underlying lung disease and did not include those with postoperative persistent air leaks [20].

The mechanism of action of OK-432 is similar to that of talc. OK-432 and talc induce an intense inflammatory response, resulting in the formation of fibrous tissue between the parietal and visceral pleura [2]. Our findings that talc resulted in lower treatment failures may be attributed to talc inducing more pleural thickening than OK-432 [24].

Exacerbation of interstitial pneumonia is a severe complication of chemical pleurodesis using OK-432, whereas acute respiratory distress syndrome is a severe complication of chemical pleurodesis using talc [25, 26]. No significant differences were observed between the two groups in terms of in-hospital mortality or incidence of interstitial lung diseases during hospitalization in the present study. Safety in talc may be similar to that in OK-432.

In the present study, the in-hospital mortality was approximately 10%. Although mortality data are scarce among patients with persistent pulmonary air leaks from spontaneous pneumothorax, a single-center retrospective Japanese study reported 15% in-hospital mortality (36/239) in elderly patients with spontaneous pneumothorax and a mean age of 79 years [27, 28]. Integrating the previous report with our findings, pneumothorax may still be a potentially fatal disease in elderly patients, necessitating an appropriate course of action.

The present study had certain limitations. First, this was a retrospective observational study. Measured confounders were adjusted for via propensity score matching; however, clinically important unmeasured confounders, such as laboratory tests and imaging findings, were not adjusted for in this study. Although we utilized propensity score matching analysis with a generalized estimating equation approach, treating individual hospitals as clusters, it is important to acknowledge that our results may have been affected by unmeasured confounders, including confounding by indications. Nevertheless, it is worth noting that we adjusted for numerous measured confounders, and the E value in our study was 1.67. As a result, it is unlikely that unmeasured confounders would have markedly altered our findings. Second, it could not be determined whether agents other than OK-432 and talc (e.g., tetracycline derivatives, autologous blood patches, and iodopovidone) were used for pleurodesis or other purposes. Therefore, other chemical agents were not considered in the analysis. Third, it was not possible to determine whether talc was used as a powder (talc poudrage) or slurry (powder mixed with saline). However, only talc slurry is commercially available in Japan. Therefore, we presumed that talc was used in the slurry form in all cases. Fourth, the precise indications for the use of OK-432 and talc in patients with pneumothorax remained unclear. During the observation period, OK-432 and talc were not approved for pleurodesis of pneumothorax in Japan. The OK-432 may have been customarily used in the public healthcare system as a well-known procedure, despite being off-label. This could explain the higher number of patients treated with OK-432 in our study. However, our study found that talc was also widely used in real-world practice in Japan for treating pneumothorax. The efficacy of talc slurry pleurodesis has been suggested in a recent phase II trial conducted in Japan, which involved patients with secondary intractable pneumothorax [29]. The use of these agents for pneumothorax in our retrospective study may have received review and approval with patients’ informed consent. However, due to the anonymous nature of the database, we have no information on that. A future prospective comparative study would be ideal to confirm our results.

## Conclusions

This retrospective nationwide cohort study demonstrated that chemical pleurodesis performed using talc reduced the frequency of treatment failure and additional pleurodesis in patients with persistent air leaks. Further prospective studies are necessary to confirm our findings.

## Supplementary Information

Below is the link to the electronic supplementary material.

Supplementary file1 (DOCX 49 KB)

## Data Availability

The datasets are not available.

## Abbreviations

CI: Confidence interval
ICD-10: International classification of diseases 10th revision
