# Supplementary material for: Comparison between OK-432 and Talc for pleurodesis in patients with persistent pulmonary air leak: a Japanese nationwide retrospective database study
Source: Gen Thorac Cardiovasc Surg. 2024 Sep 26;73(6):428–35. doi: 10.1007/s11748-024-02088-w (PMC12095331; doi:10.1007/s11748-024-02088-w)
Supplement: Supplementary file 1 — Supplementary file1 (DOCX 49 KB) [file 11748_2024_2088_MOESM1_ESM.docx]

**Supplementary Table 1**. ICD-10 codes used to define each comorbidity

| Chronic obstructive pulmonary disease | J43, J44 |
| --- | --- |
| Asthma | J45 |
| Interstitial pneumonia | J84, J99 |
| Fungal lung disease | B371, B380, B381, B382, B390, B391, B392, B400, B401, B402, B410, B420, B440, B441, B450, B460 |
| Bronchiectasis & nontuberculous mycobacteria of the lungs | J47, A310 |
| Empyema | J86 |
| Lung cancer | C34, C780 |
| Chronic respiratory failure | J961 |
| Cardiovascular disease | I20-I25, I42, I50 |
| Kidney failure | N17-N19 |
| Liver failure | K70-K77 |
| Diabetes mellites | E10-E14 |
| Autoimmune disease | M05-M09, M30-M36, K50-K51, |
| Dementia | F00-F03, G30 |

**Supplementary Table 2**. Patient characteristics at the time of admission including patients hospitalized for pneumothorax between January 2014 and March 2022

| Variables | Unmatched | | | Matched | | |
| --- | --- | --- | --- | --- | --- | --- |
|  | OK-432 (n=2,269) | Talc (n=597) | ASD | OK-432 (n=2,365) | Talc (n=591) | ASD |
| Age, years, mean (SD) | 72.1 (12.6) | 74.3 (11.1) | 18.5 | 73.8 (11.1) | 74.2 (11.1) | 3.3 |
| Male, n (%) | 2,002 (88.2%) | 494 (82.7%) | 15.6 | 1,995 (84.4%) | 491 (83.1%) | 3.5 |
| BMI, kg/m^2^, n (%) |  |  |  |  |  |  |
| <18.50 | 836 (36.8%) | 192 (32.2%) | 9.9 | 733 (31.0%) | 191 (32.3%) | 2.8 |
| 18.50–24.99 | 1,111 (49.0%) | 285 (47.7%) | 2.5 | 1,150 (48.6%) | 285 (48.2%) | 0.8 |
| 25.00–29.99 | 144 (6.3%) | 46 (7.7%) | 5.3 | 183 (7.7%) | 45 (7.6%) | 0.5 |
| ≥30.00 | 14 (0.6%) | 2 (0.3%) | 4.1 | 7 (0.3%) | 2 (0.3%) | 0.8 |
| Missing data | 164 (7.2%) | 72 (12.1%) | 16.4 | 292 (12.3%) | 68 (11.5%) | 2.6 |
| Smoking history, n (%) |  |  |  |  |  |  |
| Nonsmoker | 573 (25.3%) | 175 (29.3%) | 9.1 | 675 (28.5%) | 172 (29.1%) | 1.2 |
| Current/past smoker | 1,413 (62.3%) | 351 (58.8%) | 7.1 | 1,411 (59.7%) | 349 (59.1%) | 1.2 |
| Missing data | 283 (12.5%) | 71 (11.9%) | 1.8 | 279 (11.8%) | 70 (11.8%) | 0.1 |
| GCS score on admission, mean (SD) | 14.8 (1.0) | 14.8 (1.0) | 2.1 | 14.8 (1.0) | 14.8 (1.0) | 2.7 |
| Barthel index on admission, n (%) |  |  |  |  |  |  |
| 0 | 196 (8.6%) | 52 (8.7%) | 0.3 | 219 (9.3%) | 51 (8.6%) | 2.2 |
| 5–50 | 340 (15.0%) | 75 (12.6%) | 7.0 | 279 (11.8%) | 75 (12.7%) | 2.7 |
| 55–95 | 488 (21.5%) | 114 (19.1%) | 6.0 | 454 (19.2%) | 114 (19.3%) | 0.2 |
| 100 | 952 (42.0%) | 241 (40.4%) | 3.2 | 961 (40.6%) | 241 (40.8%) | 0.3 |
| Missing | 293 (12.9%) | 115 (19.3%) | 17.3 | 452 (19.1%) | 110 (18.6%) | 1.3 |
| Charlson comorbidity index, n (%) |  |  |  |  |  |  |
| 0 | 635 (28.0%) | 115 (19.3%) | 20.6 | 543 (23.0%) | 115 (19.5%) | 8.6 |
| 1 | 678 (29.9%) | 116 (19.4%) | 24.4 | 498 (21.1%) | 116 (19.6%) | 3.6 |
| 2 | 389 (17.1%) | 139 (23.3%) | 15.3 | 430 (18.2%) | 138 (23.4%) | 12.8 |
| 3 | 272 (12.0%) | 62 (10.4%) | 5.1 | 238 (10.1%) | 62 (10.5%) | 1.4 |
| ≥4 | 295 (13.0%) | 165 (27.6%) | 37.0 | 656 (27.7%) | 160 (27.1%) | 1.5 |
| Lung disease, n (%) |  |  |  |  |  |  |
| Chronic obstructive pulmonary disease | 856 (37.7%) | 178 (29.8%) | 16.8 | 683 (28.9%) | 178 (30.1%) | 2.7 |
| Asthma | 121 (5.3%) | 23 (3.9%) | 7.1 | 84 (3.6%) | 23 (3.9%) | 1.8 |
| Interstitial pneumonia | 182 (8.0%) | 75 (12.6%) | 15.0 | 296 (12.5%) | 73 (12.4%) | 0.5 |
| Fungal lung disease | 26 (1.1%) | 6 (1.0%) | 1.4 | 20 (0.8%) | 6 (1.0%) | 1.8 |
| Bronchiectasis & NTM of the lungs | 19 (0.8%) | 6 (1.0%) | 1.8 | 21 (0.9%) | 6 (1.0%) | 1.3 |
| Empyema | 5 (0.2%) | 5 (0.8%) | 8.5 | 13 (0.5%) | 4 (0.7%) | 1.6 |
| Lung cancer | 376 (16.6%) | 138 (23.1%) | 16.5 | 559 (23.6%) | 137 (23.2%) | 1.1 |
| Chronic respiratory failure | 132 (5.8%) | 41 (6.9%) | 4.3 | 142 (6.0%) | 40 (6.8%) | 3.1 |
| Cardiovascular disease, n (%) | 144 (6.3%) | 39 (6.5%) | 0.8 | 138 (5.8%) | 39 (6.6%) | 3.2 |
| Kidney failure, n (%) | 41 (1.8%) | 11 (1.8%) | 0.3 | 49 (2.1%) | 11 (1.9%) | 1.5 |
| Liver failure, n (%) | 36 (1.6%) | 12 (2.0%) | 3.2 | 56 (2.4%) | 12 (2.0%) | 2.3 |
| Diabetes mellitus, n (%) | 307 (13.5%) | 101 (16.9%) | 9.4 | 436 (18.4%) | 99 (16.8%) | 4.4 |
| Autoimmune disease, n (%) | 45 (2.0%) | 10 (1.7%) | 2.3 | 33 (1.4%) | 10 (1.7%) | 2.4 |
| Dementia, n (%) | 85 (3.7%) | 26 (4.4%) | 3.1 | 101 (4.3%) | 25 (4.2%) | 0.2 |
| ICU or HCU admission, n (%) | 64 (2.8%) | 17 (2.8%) | 0.2 | 60 (2.5%) | 17 (2.9%) | 1.0 |
| Teaching hospital admission, n (%) | 1,946 (85.8%) | 515 (86.3%) | 1.4 | 2,029 (858%) | 510 (86.3%) | 1.4 |
| Treatment within two days of admission day, n (%) |  |  |  |  |  |  |
| Oxygenation | 1,264 (55.7%) | 330 (55.3%) | 0.9 | 1,272 (53.8%) | 325 (55.0%) | 2.4 |
| Mechanical ventilation | 64 (2.8%) | 17 (2.8%) | 0.2 | 72 (3.0%) | 17 (2.9%) | 1.0 |
| Renal replacement therapy | 8 (0.4%) | 0 (0.0%) | 8.4 | 0 (0.0%) | 0 (0.0%) | 0.0 |
| Antibiotics | 552 (24.3%) | 150 (25.1%) | 1.8 | 601 (25.4%) | 149 (25.2%) | 0.5 |
| Antifungal drugs | 25 (1.1%) | 9 (1.5%) | 3.6 | 30 (1.3%) | 9 (1.5%) | 2.2 |
| Steroids | 265 (11.7%) | 92 (15.4%) | 10.9 | 358 (15.1%) | 91 (15.4%) | 0.7 |

ASD, absolute standardized difference; BMI, body mass index; GCS, Glasgow Coma Scale; HCU, high care unit; ICU, intensive care unit; NTM, nontuberculous mycobacteria; SD, standard deviation.

The total number of etiologies does not add up to 100% as more than one cause was assigned to a single patient.

**Supplementary Table 3**. Comparison of outcomes between the groups including patients hospitalized for pneumothorax between January 2014 and March 2022 in the propensity score matched cohort

|  | OK-432 (n=2,365) | Talc (n=591) | Risk difference | *P* |
| --- | --- | --- | --- | --- |
| Primary outcome |  |  |  |  |
| Treatment failure, n (%) | 912 (38.6%) | 186 (31.5%) | -7.9 (-13.2 to -2.5) | 0.004 |
| Surgical procedure after pleurodesis use | 197 (8.3%) | 32 (5.4%) | -3.5 (-6.0 to -1.0) | 0.005 |
| Bronchial intervention after pleurodesis use | 18 (0.8%) | 9 (1.5%) | 1.0 (-0.3 to 2.2) | 0.132 |
| Additional pleurodesis use | 780 (33.0%) | 171 (28.9%) | -4.7 (-9.9 to 0.6) | 0.085 |
| Secondary outcome |  |  |  |  |
| In-hospital mortality, n (%) | 178 (7.5%) | 53 (9.0%) | 2.5 (-1.1 to 6.1) | 0.180 |
| Length of hospital stay, days, mean (SD) | 18.8 (18.3) | 19.4 (17.4) | 0.8 (-1.0 to 2.6) | 0.378 |
| Readmission within 30 days, n (%) | 123 (5.2%) | 43 (7.3%) | 2.0 (-0.6 to 4.7) | 0.130 |
| Incidence of interstitial lung diseases, n (%) | 13 (0.5%) | 3 (0.5%) | 0.2 (-0.5 to 1.0) | 0.592 |

SD, standard deviation

**Supplementary Table 4**. Patient characteristics at the time of admission excluding patients with lung tumors

| Variables | Unmatched | | | Matched | | |
| --- | --- | --- | --- | --- | --- | --- |
|  | OK-432 (n=2,960) | Talc (n=490) | ASD | OK-432 (n=1,952) | Talc (n=488) | ASD |
| Age, years, mean (SD) | 71.9 (13.3) | 74.9 (10.8) | 24.4 | 74.5 (11.3) | 74.8 (10.8) | 2.7 |
| Male, n (%) | 2,628 (88.8%) | 412 (84.1%) | 13.8 | 1,657 (84.9%) | 410 (84.0%) | 2.4 |
| BMI, kg/m^2^, n (%) |  |  |  |  |  |  |
| <18.50 | 1,139 (38.5%) | 161 (32.9%) | 11.8 | 625 (32.0%) | 161 (33.0%) | 2.1 |
| 18.50–24.99 | 1,380 (46.6%) | 228 (46.5%) | 0.2 | 908 (46.5%) | 228 (46.7%) | 0.4 |
| 25.00–29.99 | 155 (5.2%) | 33 (6.7%) | 6.3 | 132 (6.8%) | 33 (6.8%) | 0.0 |
| ≥30.00 | 15 (0.5%) | 1 (0.2%) | 5.1 | 7 (0.4%) | 1 (0.2%) | 2.9 |
| Missing data | 271 (9.2%) | 67 (13.7%) | 14.2 | 280 (14.3%) | 65 (13.3%) | 3.0 |
| Smoking history, n (%) |  |  |  |  |  |  |
| Nonsmoker | 820 (27.7%) | 140 (28.6%) | 1.9 | 568 (29.1%) | 140 (28.7%) | 0.9 |
| Current/past smoker | 1,790 (60.5%) | 296 (60.4%) | 0.1 | 1,196 (61.3%) | 294 (60.2%) | 2.1 |
| Missing data | 350 (11.8%) | 54 (11.0%) | 2.5 | 188 (9.6%) | 54 (11.1%) | 4.7 |
| GCS score on admission, mean (SD) | 14.8 (1.0) | 14.8 (1.1) | 2.5 | 14.8 (1.1) | 14.8 (1.1) | 0.2 |
| Barthel index on admission, n (%) |  |  |  |  |  |  |
| 0 | 271 (9.2%) | 44 (9.0%) | 0.6 | 204 (10.5%) | 44 (9.0%) | 4.8 |
| 5–50 | 445 (15.0%) | 64 (13.1%) | 5.7 | 248 (12.7%) | 64 (13.1%) | 1.2 |
| 55–95 | 647 (21.9%) | 94 (19.2%) | 6.6 | 356 (18.2%) | 94 (19.3%) | 2.6 |
| 100 | 1,188 (40.1%) | 189 (38.6%) | 3.2 | 752 (38.5%) | 189 (38.7%) | 0.4 |
| Missing | 409 (13.8%) | 99 (20.2%) | 17.1 | 392 (20.1%) | 97 (19.9%) | 0.5 |
| Charlson comorbidity index, n (%) |  |  |  |  |  |  |
| 0 | 948 (32.0%) | 123 (25.1%) | 15.4 | 519 (26.6%) | 123 (25.2%) | 3.2 |
| 1 | 1,153 (39.0%) | 134 (27.3%) | 24.8 | 569 (29.1%) | 134 (27.5%) | 3.8 |
| 2 | 357 (12.1%) | 94 (19.2%) | 19.7 | 280 (14.3%) | 93 (19.1%) | 12.7 |
| 3 | 274 (9.3%) | 43 (8.8%) | 1.7 | 184 (9.4%) | 43 (8.8%) | 2.1 |
| ≥4 | 228 (7.7%) | 96 (19.6%) | 35.2 | 400 (20.5%) | 95 (19.5%) | 2.6 |
| Lung disease, n (%) |  |  |  |  |  |  |
| Chronic obstructive pulmonary disease | 1,274 (43.0%) | 166 (33.9%) | 18.9 | 674 (34.5%) | 166 (34.0%) | 1.1 |
| Asthma | 171 (5.8%) | 22 (4.5%) | 5.8 | 99 (5.1%) | 22 (4.5%) | 2.6 |
| Interstitial pneumonia | 240 (8.1%) | 64 (13.1%) | 16.2 | 244 (12.5%) | 62 (12.7%) | 0.6 |
| Fungal lung disease | 31 (1.0%) | 6 (1.2%) | 1.7 | 17 (0.9%) | 6 (1.2%) | 3.5 |
| Bronchiectasis & NTM of the lungs | 31 (1.0%) | 6 (1.2%) | 1.7 | 23 (1.2%) | 6 (1.2%) | 0.5 |
| Empyema | 8 (0.3%) | 5 (1.0%) | 9.4 | 20 (1.0%) | 4 (0.8%) | 2.1 |
| Lung cancer | 0 (0.0%) | 0 (0.0%) | 0.0 | 0 (0.0%) | 0 (0.0%) | 0.0 |
| Chronic respiratory failure | 197 (6.7%) | 33 (6.7%) | 0.3 | 142 (7.3%) | 32 (6.6%) | 2.8 |
| Cardiovascular disease, n (%) | 185 (6.3%) | 32 (6.5%) | 1.1 | 117 (6.0%) | 32 (6.6%) | 2.3 |
| Kidney failure, n (%) | 56 (1.9%) | 10 (2.0%) | 1.1 | 37 (1.9%) | 10 (2.0%) | 1.1 |
| Liver failure, n (%) | 43 (1.5%) | 11 (2.2%) | 5.9 | 47 (2.4%) | 11 (2.3%) | 1.0 |
| Diabetes mellitus, n (%) | 369 (12.5%) | 83 (16.9%) | 12.7 | 299 (15.3%) | 82 (16.8%) | 4.0 |
| Autoimmune disease, n (%) | 59 (2.0%) | 8 (1.6%) | 2.7 | 37 (1.9%) | 8 (1.6%) | 1.9 |
| Dementia, n (%) | 97 (3.3%) | 25 (5.1%) | 9.1 | 81 (4.1%) | 24 (4.9%) | 3.7 |
| ICU or HCU admission, n (%) | 79 (2.7%) | 14 (2.9%) | 1.1 | 64 (3.4%) | 14 (2.9%) | 2.4 |
| Teaching hospital admission, n (%) | 2,583 (87.3%) | 433 (88.4%) | 3.4 | 1,704 (87.3%) | 432 (88.5%) | 3.8 |
| Treatment within two days of admission day, n (%) |  |  |  |  |  |  |
| Oxygenation | 1,713 (57.9%) | 281 (57.3%) | 1.1 | 1,121 (57.4%) | 279 (57.2%) | 0.5 |
| Mechanical ventilation | 80 (2.7%) | 14 (2.9%) | 0.9 | 65 (3.3%) | 14 (2.9%) | 2.7 |
| Renal replacement therapy | 11 (0.4%) | 0 (0.0%) | 8.6 | 0 (0.0%) | 0 (0.0%) | 0.0 |
| Antibiotics | 776 (26.2%) | 130 (26.5%) | 0.7 | 553 (28.3%) | 129 (26.4%) | 4.3 |
| Antifungal drugs | 26 (0.9%) | 7 (1.4%) | 5.2 | 20 (1.0%) | 7 (1.4%) | 3.7 |
| Steroids | 327 (11.0%) | 70 (14.3%) | 9.7 | 269 (13.8%) | 69 (14.1%) | 1.0 |

ASD, absolute standardized difference; BMI, body mass index; GCS, Glasgow Coma Scale; HCU, high care unit; ICU, intensive care unit; NTM, nontuberculous mycobacteria; SD, standard deviation.

The total number of etiologies does not add up to 100% as more than one cause was assigned to a single patient.

**Supplementary Table 5**. Comparison of outcomes between the groups excluding patients with lung tumors in the propensity score matched cohort

|  | OK-432 (n=1,952) | Talc (n=488) | Risk difference | *P* |
| --- | --- | --- | --- | --- |
| Primary outcome |  |  |  |  |
| Treatment failure, n (%) | 763 (39.1%) | 151 (30.9%) | -9.0 (-14.3 to -3.8) | 0.001 |
| Surgical procedure after pleurodesis use | 135 (6.9%) | 23 (4.7%) | -2.9 (-5.3 to -0.5) | 0.020 |
| Bronchial intervention after pleurodesis use | 13 (0.7%) | 8 (1.6%) | 1.2 (-0.1 to 2.4) | 0.068 |
| Additional pleurodesis use | 678 (34.7%) | 139 (28.5%) | -6.8 (-12.1 to -1.4) | 0.013 |
| Secondary outcome |  |  |  |  |
| In-hospital mortality, n (%) | 154 (7.9%) | 38 (7.8%) | 0.3 (-3.2 to 3.7) | 0.885 |
| Length of hospital stay, days, mean (SD) | 20.2 (19.0) | 20.0 (18.4) | 0.4 (-1.7 to 2.5) | 0.677 |
| Readmission within 30 days, n (%) | 101 (5.2%) | 36 (7.4%) | 2.2 (-0.4 to 4.9) | 0.102 |
| Incidence of interstitial lung diseases, n (%) | 11 (0.6%) | 2 (0.4%) | -0.1 (-0.8 to 0.6) | 0.828 |

SD, standard deviation
